# Higher Risk of Stroke Is Correlated With Increased Opportunistic Pathogen Load and Reduced Levels of Butyrate-Producing Bacteria in the Gut

**DOI:** 10.3389/fcimb.2019.00004

**Published:** 2019-02-04

**Authors:** Xiuli Zeng, Xuxuan Gao, Yu Peng, Qiheng Wu, Jiajia Zhu, Chuhong Tan, Genghong Xia, Chao You, Ruoting Xu, Suyue Pan, Hongwei Zhou, Yan He, Jia Yin

**Affiliations:** ^1^Department of Neurology, Nanfang Hospital, Southern Medical University, Guangzhou, China; ^2^State Key Laboratory of Organ Failure Research, Microbiome Medicine Center, Division of Laboratory Medicine, Zhujiang Hospital, Southern Medical University, Guangzhou, China

**Keywords:** stroke risk, fecal, microbiota, 16S rRNA, short-chain fatty acids

## Abstract

**Objective:** Gut microbiota is a newly identified risk factor for stroke, and there are no large prospective studies linking the baseline gut microbiome to long-term risk of stroke. We present here the correlation between the gut microbiota and stroke risk in people with no prior stroke history.

**Methods:** A total of 141 participants aged ≥60 years without prior history of stroke were recruited and divided into low-risk, medium-risk, and high-risk groups based on known risk factors and whether they were suffering from chronic diseases. The composition of their gut microbiomes was compared using 16S rRNA gene amplicon next-generation-sequencing and Quantitative Insights into Microbial Ecology (QIIME) analysis. Levels of fecal short-chain fatty acids were measured using gas chromatography.

**Results:** We found that opportunistic pathogens (e.g., Enterobacteriaceae and Veillonellaceae) and lactate-producing bacteria (e.g., *Bifidobacterium* and *Lactobacillus*) were enriched, while butyrate-producing bacteria (e.g., Lachnospiraceae and Ruminococcaceae) were depleted, in the high-risk group compared to the low-risk group. Butyrate concentrations were also lower in the fecal samples obtained from the high-risk group than from the low-risk group. The concentrations of other short-chain fatty acids (e.g., acetate, propionate, isobutyrate, isovalerate, and valerate) in the gut were comparable among the three groups.

**Conclusion:** Participants at high risk of stroke were characterized by the enrichment of opportunistic pathogens, low abundance of butyrate-producing bacteria, and reduced concentrations of fecal butyrate. More researches into the gut microbiota as a risk factor in stroke should be carried out in the near future.

## Introduction

Stroke is a serious public health problem worldwide and is ranked as the second-most fatal disease (GBD 2015 Mortality and Causes of Death Collaborators, 2016). In recent years, the annual incidence of first-time ischemic strokes in China has increased by 8.3% (Guan et al., 2017), and the disease now represents a significant public health burden. Effective prevention, focusing on modifying the factors known to be associated with stroke, remains the best approach for reducing disease risk (Meschia et al., 2014; Powers et al., 2018). Ninety percent of stroke cases may be linked to behavioral factors such as smoking, poor diet, and low physical activity or to metabolic factors such as hypertension, obesity, diabetes mellitus, and high total cholesterol. Identifying novel risk factors is critical and may contribute to the development of more effective intervention methods.

The microbiome of the gut has recently received a fair amount of attention as a potential risk factor in stroke. Accumulating evidence has demonstrated that the gut microbiota may be closely correlated with the pathogenesis of clinical risk factors (e.g., hypertension, diabetes mellitus, and obesity) associated with stroke (Ley et al., 2006; Sato et al., 2014; Santisteban et al., 2017). Aberrant gut microbiota from hypertensive human donors contributes to the development of hypertension in the germ-free mice by fecal transplantation (Li et al., 2017). Additionally, higher Trimethylamine N-oxide levels, a known atherogenic gut derived metabolite, were demonstrated to be related with increased risk of stroke in hypertensive patients (Nie et al., 2018). A previous clinical study described the dysbiosis of gut microbiota in patients with acute ischemic stroke as well, with Enterobacteriaceae being particularly enriched in this instance (Yin et al., 2015). Despite the link suggested by the above studies, it remains unclear whether the dysbacteriosis seen in acute stroke patients was preexisting prior to the stroke. Because of the difficulty of predicting the exact time and place a stroke occurs, it would be impractical to obtain stool samples from a patient beforehand. Regardless, the questions of the microbial composition in the gut of persons about to suffer a stroke and whether it is similar to that of patients immediately following an acute stroke are nonetheless important.

The Stroke Screening Survey is a vitally important method widely used by physicians to determine stroke risk in China (Guan et al., 2017; Wang, W. et al., 2017; Wang et al., 2018). Based on the presence of risk factors associated with stroke and chronic diseases, the survey divides participants into three major risk categories (Mi et al., 2016; Wang et al., 2018). The present study investigates the correlation between gut microbiota and stroke risk. We compared the microbial characteristics, including microbial structure, abundance of functional groups of bacteria, and the levels of gut-microbiota-derived metabolites, of participants from different risk categories, with the objective of identifying the role of the gut microbiome in stroke.

## Materials and Methods

### Study Participants

**Figure 1:**
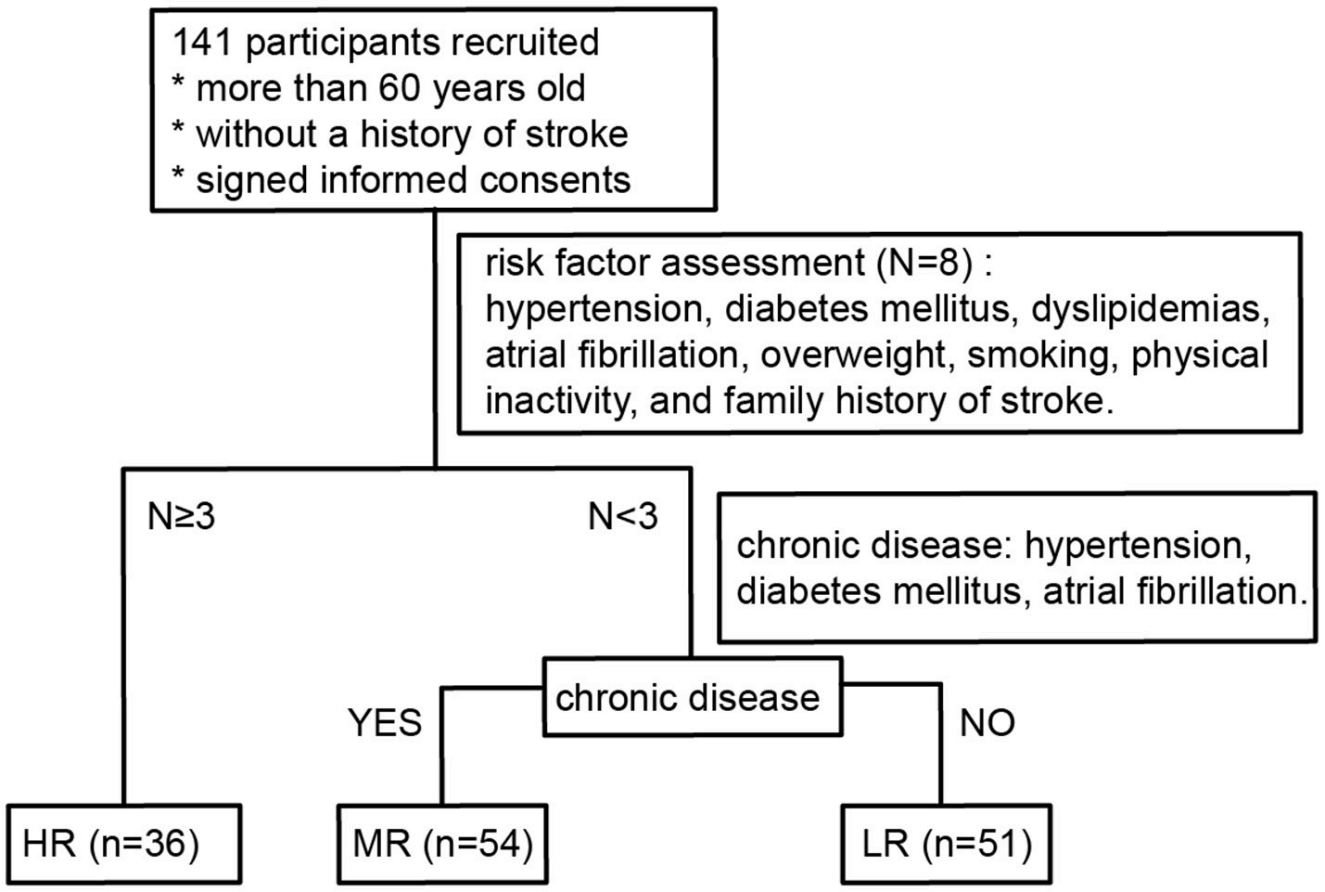
Flow diagram describing the risk stratification process for all participants. LR, low-risk group; MR, medium-risk group; HR, high-risk group.

Participants who had recently retired from positions at the Yanling Hospital or who had lived in the Bureau of Reclamation in Guangdong Province were recruited to the study between November 2016 and January 2017. All participants were Han Chinese. They had lived in Guangzhou city for more than 5 years and had been there for more than 10 months every year, without cross-border tourism recently. Individuals who had taken oral antibiotics or prebiotics in the 3 months prior to the recruitment phase or who had undergone surgery for intestinal tumors were excluded from the study. Persons younger than 60 years old or who had prior histories of myocardial infarction or stroke were also not enrolled in the study. One hundred and forty-one participants (58 males, 83 females; age range 60–88, mean age 70.9) were recruited in total. All of them were subjected to risk factor evaluation (Wang et al., 2018) following the protocols formulated by the National Health and Family Planning Commission of Stroke Screening and Prevention Project. A flow chart detailing the classification process is shown in Figure 1.

### Risk Factor Assessment

The risk factors (RFs) assessed were hypertension, diabetes mellitus, dyslipidemias, atrial fibrillation, overweight, smoking, physical inactivity, and family history of stroke. RF1: Hypertension was defined by a history of high blood pressure (or documented systolic blood pressure ≥140 mmHg or diastolic blood pressure ≥90 mmHg) reported by the participant or by the use of anti-hypertensive medicines. RF2: Atrial fibrillation, as reported by the participant or confirmed by recent electrocardiogram. RF3: Diabetes mellitus, as defined by former diagnosis, treatment with insulin/oral hypoglycemic medications, or fasting plasma glucose ≥7.0 mmol/L. RF4: Dyslipidemias, defined as having one or more of the following results: total cholesterol ≥6.22 mmol/L, serum triglycerides ≥2.26 mmol/L, high-density lipoprotein cholesterol <1.04 mmol/L, low-density lipoprotein ≥4.14 mmol/L, or current use of statins. RF5: Smoking, defined as current smoking status. RF6: Physical inactivity, defined by physical exercise <3 times a week for <30 min in duration each time (industrial and agricultural labor was considered as physical exercise). RF7: Overweight, defined by a body mass index ≥26 kg/m^2^. Body mass index was calculated as body weight (in kg) divided by the square of height (in m; kg/m^2^). RF8: Family history of stroke was self-reported by participants themselves.

The high-risk (HR) group was defined as participants with three or more risk factors. Participants with less than three risk factors were divided into two groups: those with chronic diseases (hypertension, diabetes mellitus, atrial fibrillation) were classified as medium-risk (MR) and those without chronic diseases were classified as low-risk (LR).

### Clinical Data and Sample Collection

Participant clinical data were collected by three trained neurologists through self-designed questionnaires. Dietary habits were recorded as vegetarian, omnivorous, and carnivorous according to the participants self-reported. The risk factors assessed in our questionnaire included behavioral factors (overweight, smoking, physical inactivity), family history of stroke, and biomedical factors (hypertension, diabetes mellitus, dyslipidemias, and atrial fibrillation). Fasting blood samples were biochemically examined in the Clinical Laboratory of Nanfang Hospital, and routine blood parameters were estimated, including blood lipid level, albumin, creatinine, serum uric acid, fasting blood glucose, and high-sensitivity C-reactive protein. Fresh fecal samples were obtained, frozen immediately at −20°C, and shipped using cold-chain transportation to the laboratory, before being stored at −80°C until further analysis.

### Fecal SCFA Detection

Fecal samples were frozen at −80°C, and 0.2 g of each sample was separated for SCFA analysis. Seven analytes were targeted for short-chain fatty acid (SCFA) analysis, including acetic acid, propionic acid, butyric acid (all provided by Dr. Ehrenstorfer, Germany), isobutyric acid (Supelco, USA), valeric acid (Nu-Chek, USA), and isovaleric acid (Sigma-Aldrich, USA). Fecal samples for SCFA analysis were frozen at −80°C within 3 h of voiding. Samples were first homogenized in 1.0 ml ultra-pure water containing an internal standard of 2,2-dimethylbutyric acid (also provided by Dr. Ehrenstorfer). The homogenate was then placed in an Eppendorf tube and centrifuged at 12,000 rpm at 4°C for 10 min. The resulting supernatant was transferred into a new tube and mixed with 10 μl 50% sulfuric acid and 0.5 g sodium sulfate (both Macklin, China), along with 2 mL analytically pure diethyl ether. The solution was vortexed for 1 min and then centrifuged for 10 min at 5,000 rpm at room temperature. The ether layer was collected for gas chromatography with mass selective detection (5977B GC/MSD, Agilent Technologies, Santa Clara, CA, USA) measurement. An HP-FFAP capillary column (30 m length, 0.25 mm internal diameter) was used for chromatographic separation, using helium as the carrier gas. The oven temperature was increased by 15°C every minute from 90 to 180°C. GC/MS data were acquired and analyzed using the MassHunter Workstation Software (Agilent Technologies) running on Windows 7 (Microsoft, Redmond, WA, USA). The concentrations of fecal SCFAs were calculated with the use of external standards and are expressed in units of micromoles per gram of wet feces. Detailed machine settings are recorded in Tables S1, S2.

### 16S rRNA Gene Amplicon Analysis

Bacterial DNA was extracted from fecal samples with the stool DNA extraction kit using a magnetic bead-based method (Shenzhen Bioeasy Biotechnology. Co., Ltd., China) according to the manufacturer's instructions. The V4 region of the bacterial 16S rRNA gene was amplified by quantitative real-time polymerase chain reaction (qPCR) with the bar-coded primers V4F (5′-GTGTGYCAGCMGCCGCGGTAA-3′) and V4R (5′-CCGGACTACNVGGGTWTCTAAT-3′) using the LightCycler® 480 II real-time fluorescence quantitative PCR system (Roche Diagnostics Ltd., Switzerland). The PCR protocol was as follows: (1) initial denaturation (94°C, 2 min); (2) PCR amplification (32 cycles; 94°C, 30 s; 52°C, 30 s; and then 72°C, 30 s); (3) melting (95°C, 5 s; 60°C, 1 min; then 95°C, continuous); (4) cooling (37°C, 30 s). PCR products were separated in a 1% agarose gel. Samples that gave rise to a visible product 290–310 bp in size were used in further experiments. Using the GeneTools Analysis Software (Version 4.03.05.0, SynGene), the PCR products were mixed in equimolar ratios, then purified using an EZNA Gel Extraction Kit (Omega, USA). Sequencing libraries were established using NEBNext® Ultra™ DNA Library Prep Kit for Illumina® (New England Biolabs, USA) according to the manufacturer's recommendations, and the index codes were added. The quality of the library was assessed using the Qubit@ 2.0 Fluorometer (Thermo Scientific) and Agilent Bioanalyzer 2100 systems. Finally, the library was sequenced on an Illumina HiSeq 2500 platform, and 250-bp paired-end reads were generated.

### Bioinformatics and Biostatistics

Depending on the overlap (~100 bp) between the two paired-end sequences, we then used SeqPrep to merge the paired-end sequences and assessed the quality of the result using the open-source software Quantitative Insights into Microbial Ecology (QIIME, version 1.9.1) (Caporaso et al., 2010). A QIIME workflow script, *split_libraries_fastq.py*, was used to check the quality of sequences, and Phred score ≥Q20 was considered to be qualified sequences. Then, we used a home-brewed script to split FASTA files according to paired-end barcode information, which should meet the following criteria: 100% matching barcode and primer, more than 200 bp after removing barcode and primer. Sequences longer than 200 bp were trimmed to 200 bp, and those shorter than 200 bp were removed. Two hundred bp at one end had the strength of good overlap effect, less computing resources and better quality of sequence. Finally, we used a QIIME workflow script, *pick_closed_reference_otus.py*, to remove chimeras, perform reference-based operational taxonomic unit (OTU) clustering and generate a BIOM file. The reference database used in our study is Greengenes 13_8 and SortMeRNA was used for clustering and classification, setting an identity cutoff to 0.97. All samples were normalized to the same level for avoiding possible errors stemming from the use of different sequencing depths. To preserve as many sequences as possible, we set 3,100 tags as normalization, and a total of 437,100 high-quality sequences were used for further analysis. The same analysis with a normalized level of 6,000 tags (Figure S1) and DADA2 (Figure S2) were done in our study, strengthening the reliability of our conclusions.

### Microbial Analysis

Alpha diversity (or the complexity within a community) was estimated by using the Chao 1 index, which is based on the richness of the sample; the observed number of species, which is a direct measure of species number; the Shannon diversity index, which is intended to represent species abundance and evenness; and a phylogenetic diversity (PD) whole tree, an alternate method that takes the phylogenetic differences between species that comprise the community into account. Beta diversity (which estimates differences between microbial communities) was analyzed using the Bray–Curtis distance approach. Principal coordinates analysis (PCoA), a dimensionality reduction method illustrating the relationship between samples based on a distance matrix, was performed to highlight the divergence between different groups using the R package “ade4.” The relative abundance of dominant taxa in each group is shown in stack bar plots using the R package “ggplot2.” To determine metagenomic biomarkers that differentiate two or more groups, linear discriminant analysis (LDA) coupled with effect size measurement (LEfSe) was performed using an online utility (http://huttenhower.sph.harvard.edu/galaxy/) (Segata et al., 2011). First, significant differential abundance between groups were detected using the non-parametric factorial Kruskal–Wallis sum-rank test; biological consistency is subsequently investigated among subclasses by the unpaired Wilcoxon rank-sum test. Finally, LDA score was used to estimate the effect size of each differentially abundant feature. The significant bacteria with LDA score ≥2.0 were diagrammed on taxonomy bar-chart plots. The relative abundance of microorganisms among three groups were described in the format of boxplots with overlaid dot plots, whose significant values were determined by Kruskal–Wallis test. The pairwise comparisons were adjusted by Bonferroni method. For microbial analysis, QIIME was additionally performed using the Adonis test as previously described (Yin et al., 2015). Only *p*-values < 0.05 (two-tailed) were considered statistically significant.

### Statistical Analysis

Data were analyzed using IBM's SPSS^®^ 22.0 statistical software package and R version 3.4.3 (https://www.r-project.org/). Continuous variables are represented in the form of median (interquartile range, IQR). The Kruskal–Wallis test, Mann–Whitney *U*-test, and Pearson chi-square test were also performed to ascertain statistical significance.

## Results

### Clinical Characteristics Among the LR, MR, and HR Groups

One hundred and forty-one participants were recruited in the present study. The characteristics of the study participants are outlined in Table 1. Based on the number of risk factors and the presence of chronic diseases, they were classified into three groups: the LR group, MR group, and HR group (see previous section for further details). We found no significant difference among the three groups in terms of age or gender (Kruskal–Wallis test, Pearson chi-square test, all *p* > 0.05). However, white blood cell, neutrophils, and red blood cell counts and high sensitivity C-reactive protein were significantly higher in the HR group than in the LR group. High-density lipoprotein cholesterol was lowest in the HR group. Dietary habit can affect the risk of stroke. In this study, 48 participants were vegetarian, 84 were omnivorous, and only 9 were carnivorous. There was no significant difference in dietary pattern among the three groups (Table 1, Pearson chi-square test, *p* = 0.648).

**Table 1:** Characteristics of the study participants.

	**LR (*n* = 51)**	**MR (*n* = 54)**	**HR (*n* = 36)**	***p*-value**
Age, year	68.76 (12.00)	72.37 (15.25)	71.58 (16.75)	0.061
Gender (M/F)	21/30	22/32	15/21	0.996
WBC, × 10^9^	5.54 (2.04)	5.88 (1.53)	6.52 (1.91)^#^^¶^	0.005*
LYM, × 10^9^	2.11 (0.82)	2.02 (0.77)	2.09 (1.05)	0.574
NEU, × 10^9^	2.95 (1.50)	3.15 (0.92)	3.75 (1.69)^#^^¶^	0.004*
MONO, × 10^9^	0.36 (0.17)	0.35 (0.15)	0.41 (0.23)	0.220
RBC, × 10^12^	4.50 (0.54)	4.48 (0.79)	4.78 (0.96)^¶^	0.031*
PLT, × 10^9^	249 (67.00)	231 (64.50)	231 (56.25)	0.319
HGB, g/L	135.0 (12.00)	136.5 (24.00)	130.5 (24.25)	0.548
BUN, mmol/L	5.10 (1.60)	5.10 (1.05)	5.35 (1.55)	0.618
CR, μmol/L	67.0 (22.00)	67.5 (23.00)	69.0 (20.50)	0.833
UA, μmol/L	363 (112.00)	337 (111.50)	346 (116.00)	0.474
ALB, g/L	41.8 (3.60)	41.5 (3.18)	41.9 (4.30)	0.492
FBG, mmol/L	4.61 (0.47)	4.69 (0.77)	4.90 (0.79)	0.097
TG, mmol/L	1.24 (0.85)	1.22 (0.61)	1.40 (0.87)	0.301
TC, mmol/L	5.47 (1.39)	5.11 (1.51)	5.09 (1.60)	0.124
HDL, mmol/L	1.33 (0.39)	1.40 (0.38)	1.12 (0.33)^#^^¶^	0.0001*
LDL, mmol/L	3.47 (1.16)	3.07 (0.97)	3.23 (1.24)	0.240
Hs-CRP, mg/L	0.86 (2.18)	0.93 (1.69)	1.46 (2.76)^#^	0.030*
Dietary habits				0.648
Vegetarian	18 (35.3%)	17 (31.5%)	13 (36.1%)	
Omnivorous	30 (58.8%)	35 (64.8%)	19 (52.8%)
Carnivorous	3 (5.9%)	2 (3.7%)	4 (11.1%)
Hypertension	0	50 (92.6%)	31 (86.1%)	NA
Diabetes mellitus	0	5 (9.3%)	8 (22.2%)	NA
Dyslipidemias	32 (62.7%)	36 (66.7%)	32 (88.9%)	NA
Smoking	3 (5.9%)	1 (1.9%)	9 (25%)	NA
Physical inactivity	13 (25.5%)	4 (7.4%)	15 (41.7%)	NA
Atrial fibrillation	0	1 (1.9%)	4 (11.1%)	NA
Family history of stroke	4 (7.8%)	0	5 (13.9%)	NA
Overweight	3 (5.9%)	0	16 (44.4%)	NA

*WBC, white blood cells; NEU, neutrophils; RBC, red blood cells; UA, uric acid; ALB, albumin; FBG, fasting blood glucose; TG, triglycerides; HDL-C, high-density lipoprotein cholesterol; Hs-CRP, high-sensitivity C-reactive protein. Data are presented as median (IQR), or n (proportion). LR, low-risk group; MR, medium-risk group; HR, high-risk group. The p-values of gender and dietary habits were determined by Pearson's chi-square test*.

# *p < 0.05 when compared with the LR group, Mann–Whitney U-test*.

¶ *p < 0.05 when compared with the MR group, Mann–Whitney U-test*.

* *p < 0.05, Kruskal–Wallis test*.

### HR Group Showed Significantly Different Composition of Gut Microbiota Compared With Other Groups

Fresh stool samples were taken from all participants after verbal and written consent was obtained. Their microbial composition in the gut was further analyzed using 16S rRNA gene amplicon next-generation-sequencing and QIIME. PD whole tree (Kruskal–Wallis test, *p* = 0.06), Shannon index (Kruskal–Wallis test, *p* = 0.25), Chao 1 index (Kruskal–Wallis test, *p* = 0.21), and Observed species (Kruskal–Wallis test, *p* = 0.07) were used to evaluate the alpha diversity of each sample. Our analysis revealed no significant differences in alpha diversity index among the three groups, but a slight decrease was observed in the HR group (Figures 4A–D).

**Figure 2:**
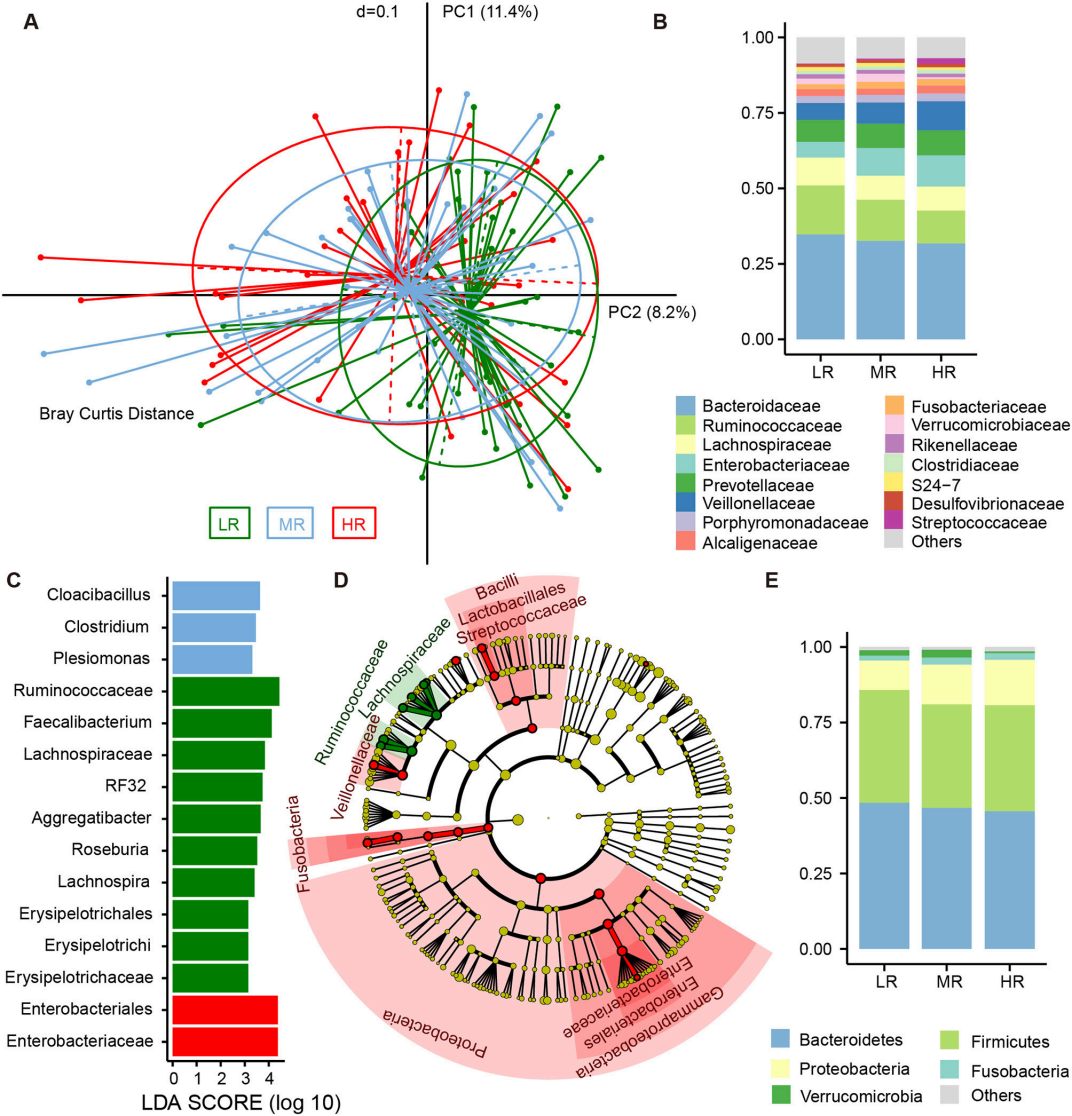
Differences in the composition of gut microbial communities between the LR (*n* = 51, green), MR (*n* = 54, blue), and HR (*n* = 36, red) groups. **(A)** Beta diversity comparison among the three groups. Principal coordinates analysis (PCoA) based on Bray–Curtis distance was used to illustrate the variations between the three groups. Average relative abundance of dominant microbes among the LR, MR, and HR groups at the family level **(B)** and phylum level **(E)**, with each color representing a taxon. **(C)** Significantly discriminative taxa among the LR, MR, and HR groups were determined using linear discriminant analysis effect size (LEfSe) analysis. **(D)** Cladograms based on LEfSe results of the LR and HR groups. LR, low-risk group; MR, medium-risk group; HR, high-risk group.

To determine the differences in microbial structure among the three groups, we performed beta diversity analysis using PCoA (an approach based on Bray–Curtis distance) within R software. Further testing was performed via Adonis. We found risk groups to be significant sources of variability in terms of gut microbiota (Adonis test, *p* = 0.03). Furthermore, a linear trend in the Bray–Curtis distance was observed from the LR group to the HR group (Figure 2A). In the pairwise comparison of Bray–Curtis distance, the LR and HR group were obviously disparate (Adonis test, *p* = 0.005), but differences between the LR and MR groups were less significant (Adonis test, *p* = 0.085).

The fecal bacteria community detected in all groups was dominated by 127 genera belonging to 5 major phyla (Figure 2E), including Firmicutes, Bacteroidetes, Proteobacteria, Verrucomicrobia, and Fusobacteria. The most predominant 15 families that made up 92.5% of the total bacteria abundance were Bacteroidaceae, Ruminococcaceae, Lachnospiraceae, Enterobacteriaceae, Prevotellaceae, Veillonellaceae, Porphyromonadaceae, Alcaligenaceae, Fusobacteriaceae, Verrucomicrobiaceae, Rikenellaceae, Clostridiaceae, S24-7, Desulfovibrionaceae, and Streptococcaceae (Figure 2B). Although these bacteria mentioned above could be detected in the gut of each subject, the relative abundance of the same microorganism in the intestines of different participants was different. For instance, the average abundance of phylum Proteobacteria was 12.41%, ranging from 2.16 to 53.52%. The average abundance of family Enterobacteriaceae was 7.74%, but range from 0.26 to 50.39%. The relative abundance of the dominant taxa at the phylum (Figure S3) and family levels (Figure S4) was illustrated in the area charts. To identify differentially abundant microbiota among the three groups, linear discriminant analysis (LDA) coupled with effect size measurement (LEfSe) was performed. With this approach, we determined that Enterobacteriaceae was enriched in the HR group, while those of the Erysipelotrichaceae subclass, the Ruminococcaceae family, and the genera *Lachnospira, Roseburia, Aggregatibacter, RF32*, and *Faecalibacterium* were mostly related to low risk of stroke (Figure 2C). LEfSe further revealed significant distinctions in bacterial taxa between the LR and HR groups: in addition to the aforementioned taxa, we saw that the relative abundance of Proteobacteria, Bacilli, Lactobacillales, Veillonellaceae, Streptococcaceae, *Megasphaera*, and *Fusobacterium* in the HR group was also higher than that in the LR group (Figure 2D).

### A Higher Abundance of Opportunistic Pathogens and Lower Abundance of Butyrate-Producing Bacteria Was Observed in the HR Group

**Figure 3:**
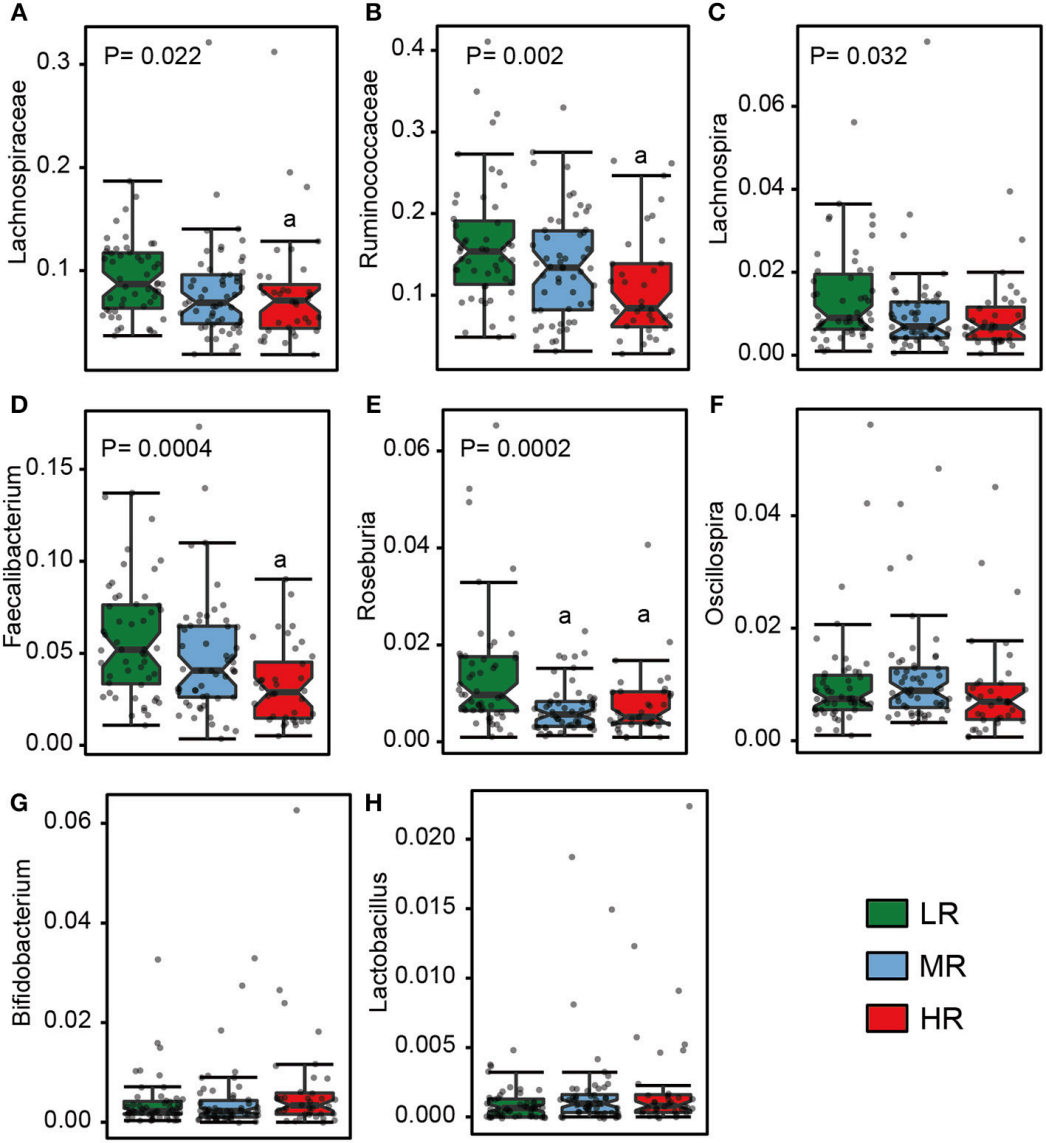
The relative abundance of butyrate- **(A–F)** and lactate-producing bacteria **(G–H)** in the gut in the LR (*n* = 51, green), MR (*n* = 54, blue), and HR groups (*n* = 36, red). The significances (*P*-value) among three groups were determined by the Kruskal–Wallis test and subsequent pairwise comparisons were adjusted by Bonferroni correction. “a” denotes instances where *p* < 0.05 when a comparison was performed with the LR group. LR, low-risk group; MR, medium-risk group; HR, high-risk group.

**Figure 4:**
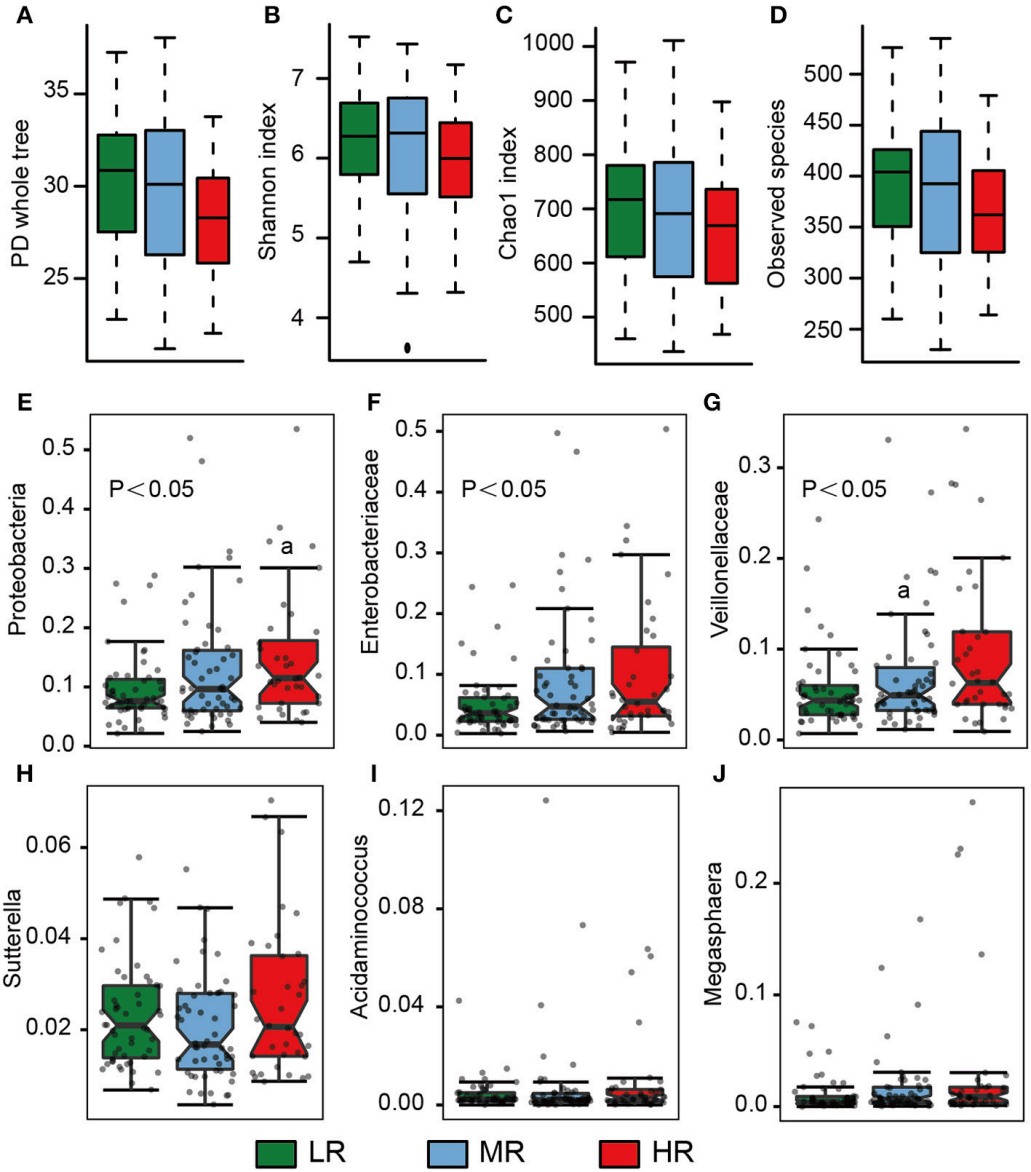
Microbial diversity and the average abundance of opportunistic pathogens among the LR (*n* = 51, green), MR (*n* = 54, blue), and HR groups (*n* = 36, red). **(A–D)** Alpha diversity among the three groups. **(E–J)** The relative abundances of opportunistic pathogens in the gut among the three groups. The significances (*P*-value) among three groups were determined by the Kruskal–Wallis test and subsequent pairwise comparisons were adjusted by Bonferroni correction. “a” denotes instances where *p* < 0.05 when a comparison was performed with the LR group. LR, low-risk group; MR, medium-risk group; HR, high-risk group. PD, phylogenetic diversity.

We also calculated the relative abundances of butyrate-producing bacteria, lactate-producing bacteria and opportunistic pathogens in the LR, MR, and HR groups. We found that, compared to the LR and MR groups, the HR group harbored lower levels of butyrate-producing bacteria belonging to Lachnospiraceae, Ruminococcaceae, *Faecalibacterium, Roseburia, Lachnospira*, and *Oscillospira* (Figures 3A–F). Furthermore, the HR group showed an increase in opportunistic bacteria, such as Proteobacteria, Enterobacteriaceae, Veillonellaceae, *Megasphaera, Veillonella, Acidaminococcus*, and *Sutterella* (Figures 4E–J). Interestingly, our results also demonstrated an increase in the average abundance of *Bifidobacterium* and *Lactobacillus* (two known probiotics) in the HR group (Figures 3G,H).

### Fecal Butyrate Concentration Was Decreased in the HR Group

We additionally measured the fecal SCFA levels in all participants enrolled in this study, including acetate, propionate, isobutyrate, butyrate, isovalerate, and valerate. Nine samples were unavailable because of the loss of frozen stool samples. Our analysis revealed that the average concentration (AC) of acetate was 82.26 μmol/g, the highest among the six SCFAs that we assayed for. Propionate ranked the second (22.09 μmol/g) and butyrate third (14.14 μmol/g; Table 2). There were no significant differences in the AC of acetate among the LR (86.10 μmol/g), MR (79.35 μmol/g), and HR groups (81.0 μmol/g) (Mann–Whitney *U*-test, *p* > 0.05). Likewise, no significant differences were observed in the AC of propionate (LR, 24.95 μmol/g; MR, 21.40 μmol/g; HR, 18.80 μmol/g), isobutyrate (LR, 1.37 μmol/g; MR, 1.50 μmol/g; HR, 1.23 μmol/g), isovalerate (LR, 0.99 μmol/g; MR, 1.15 μmol/g; HR, 0.89 μmol/g), or valerate (LR, 1.56 μmol/g; MR, 1.45 μmol/g; HR, 1.53 μmol/g) among the three groups (Mann–Whitney *U*-test, all *p* > 0.05). Only the AC of butyrate was significantly different, being lower in the MR (11.22 μmol/g) and HR groups (13.45 μmol/g) compared to the LR group (17.62 μmol/g) (Mann–Whitney *U*-test, *p* = 0.003, *p* = 0.04, respectively).

**Table 2:** Concentration of fecal SCFAs in study participants.

	**LR (*n* = 49)**	**MR (*n* = 51)**	**HR (*n* = 32)**	***p*-value**
Acetate, μmol/g	83.15 (55.90)	70.43 (63.59)	76.51 (51.41)	0.674
Propionate, μmol/g	21.31 (19.45)	19.62 (12.14)	16.21 (17.21)	0.348
Isobutyrate, μmol/g	0.83 (1.45)	1.12 (1.84)	0.74 (2.03)	0.527
Butyrate, μmol/g	17.94 (15.61)	7.79 (10.44)^#^	10.17 (11.88)^#^	0.002*
Isovalerate, μmol/g	0.51 (1.29)	0.80 (1.72)	0.23 (1.36)	0.311
Valerate, μmol/g	1.48 (2.21)	1.17 (2.06)	1.53 (2.40)	0.824

*Data are presented as median (IQR). SCFAs, short-chain fatty acids. LR, low-risk group; MR, medium-risk group; HR, high-risk group*.

# *p < 0.05 compared with the LR group, Mann–Whitney U-test*.

* *p < 0.05, Kruskal–Wallis test*.

### The Gut Microbial Compositions of Different Risk Groups With or Without Medication Use

Medication can alter gut microbiota composition and potentially confound our analysis (Forslund et al., 2015; Maier et al., 2018). In our study, 78 participants were taking at least one kind of medicine, including statins (e.g., atorvastatin, rosuvastatin), antihypertensive drug (e.g., amlodipine, nifedipine), and hypoglycemic agents (e.g., metformin, acarbose). To determine the effects of oral medicat ion on gut microbial composition, we separately compared the gut microbiota of different risk groups with or without medication use. In the microbial comparison of different risk groups without any medicine used, we found that the results accorded with those of the total participants in the alpha diversity and microbial composition (Figures S5A–F). LEfSe analysis determined that several opportunistic pathogens (e.g., Betaproteobacteria, Desulfovibrionaceae, and Actinomycetaceae) were enriched in the HR group, while *Faecalibacterium* and Peptostreptococcaceae were mostly related to lower risk of stroke (Figure S5G). Interestingly, in the microbial comparison of different risk groups with at least one kind of medicine used, we also found similar trends in the alpha diversity and microbial composition (Figures S6A–F). LEfSe analysis showed that the abundance of opportunistic pathogens (e.g., Veillonellaceae) was significantly higher in the HR group, while Ruminococcaceae and Lachnospiraceae were mostly higher in the LR group (Figure S6G). These results indicate that the gut microbiome features of each risk group were not due to medication usage.

## Discussion

The present study focused mainly on the correlation between the gut microbiome and stroke risk using a risk stratification approach. One hundred and forty-one participants with various levels of stroke risk, who were all ≥60 years of age and who did not have a prior history of stroke, were recruited to the study, and the microbial characteristics of their fecal samples were analyzed using a number of approaches. The key finding of this study is that individuals at a high risk of stroke had significantly elevated levels of opportunistic pathogens in their guts compared to those at low risk, with Enterobacteriaceae showing the greatest difference. Further, a lower concentration in butyrate-producing bacteria and fecal butyrate concentrations were also associated with a high risk of stroke.

Age is an important risk factor for stroke. Based on World Population Aging 2013 published by the United Nations, the proportion of the population aged ≥60 years in China will increase from 12.4 to 28.1% between 2010 and 2040 (United Nations, 2013), and the most recent estimates indicate that more than 80% of stroke-associated mortality instances in China are in persons of this age group (Wang, Z. et al., 2017). The above illustrates why the issue of stroke among the elderly deserves greater attention. An individual's stroke risk profile takes into consideration a number of factors, including age, gender, smoking, and the presence of comorbidities such as hypertension, diabetes mellitus, and prior cardiovascular disease. Accounting for these, a 10-year stroke risk score can be calculated, and this profile may further provide suggestions on how to reduce stroke risk through behavior modification (D'Agostino et al., 1994). However, this approach has not entered common use among primary-care physicians in developing regions, who have thus far relied on the Stroke Screening Survey instead, which has become the primary means of conducting stroke risk assessment in basic-level hospitals in China. The Survey allows for the convenient stratification of persons into HR, MR, and LR groups according to the aforementioned indices (Guan et al., 2017; Wang, W. et al., 2017; Wang et al., 2018).

The brain plays an important role in governing and coordinating systemic homeostasis. However, the role of gut health in brain dysfunction has been neglected until the mystery of the cross-talk between gut and brain started to be uncovered (Rhee et al., 2009; Bercik et al., 2011). Recent studies have suggested that the gut microbiome may also experience alterations following stroke (Yin et al., 2015), and may be, in itself, a risk factor for stroke. Our finding that the levels of opportunistic pathogens (e.g., Proteobacteria, Bacilli, Enterobacteriaceae) were more increased in the higher-risk group and that the levels of butyrate-producing bacteria (e.g., Lachnospiraceae, Ruminococcaceae) were less abundant in the same group would appear to indicate that the microflora of the gut should at least be considered in evaluations of stroke risk. The proliferation of Proteobacteria (mainly Enterobacteriaceae) in the gut is regarded as a marker for epithelial dysfunction and as an indicator of an unstable microbial community structure (Litvak et al., 2017). In healthy individuals, commensal Enterobacteriaceae are benign (Winter et al., 2013), but when homeostasis is disrupted by environmental or host factors, such as a low-fiber diet or acute or chronic inflammation, the sudden growth of the Enterobacteriaceae population can lead to the exacerbation of inflammation or invasion by exogenous pathogens (Shin et al., 2015). Microbial dysbiosis in conditions such as inflammatory bowel diseases (e.g., Crohn's disease, ulcerative colitis) and chronic kidney disease, both of which have been associated with an increase in stroke risk (Lee et al., 2010; Kristensen et al., 2014; Xiao et al., 2015), are likewise characterized by the enrichment of Enterobacteriaceae. In our study, the relative abundance of Proteobacteria and Enterobacteriaceae, the latter of which might be a potential risk factor for high risk of stroke, increased in the HR group. The levels of other pathogenic bacteria, such as *Sutterella, Megasphaera, Veillonella*, and *Acidaminococcus*, were also increased in the HR group. *Sutterella* was found to be enriched in the gut of patients with metabolic syndrome (Lim et al., 2017), prediabetes (Allin et al., 2018), and Autism (Williams et al., 2012). *Megasphaera, Veillonella*, and *Acidaminococcus* belong to the family Veillonellaceae, which is known for producing succinate, a potential microbe-derived metabolite associated with an increased risk of cardiovascular disease (Serena et al., 2018). The succinate concentration in the stool samples of different risk groups could not be detected in the present study. These evidences strongly suggest that the increased abundance of opportunistic pathogens could be associated with increased stroke risk.

Butyrate, principally derived from the enteric microbiome, plays an important role in human health, being capable of modulating host physiology, energy metabolism, and immune function and acting as a neuroprotectant (Donohoe et al., 2011; Rane et al., 2012; Stilling et al., 2016; Rose et al., 2018). Part of the reason that a high-fiber diet is recommended, and why it is thought to reduce stroke risk, may be related to the fact that it promotes the production of butyrate (Bourassa et al., 2016). In particular, gavage with butyrate has been observed to alleviate diet-induced obesity, hyperinsulinemia, hypertriglyceridemia in mice, mainly by reducing appetite and activating brown adipose tissue (Li et al., 2018), and increasing the consumption of dietary fiber has a similarly salutary effect on diabetes. Butyrate additionally suppress the synthesis of nitrate and nitride, which may act as a respiratory source for Enterobacteriaceae (Byndloss et al., 2017). In contrast to opportunistic pathogens, butyrate-producing bacteria may ameliorate certain conditions known to be linked to increased stroke risk, such as hypertension, diabetes mellitus, and obesity (Qin et al., 2012; Li et al., 2017; Gao et al., 2018), consistent with our results. The levels of the known butyrate-producing bacteria Lachnospiraceae, Ruminococcaceae, *Faecalibacterium, Roseburia, Lachnospira*, and *Butyricicoccus* were significantly lower in the HR group. Butyrate concentration was likewise significantly lower in that group. The facts that butyrate may have a neuroprotective effect and that it could decrease stroke risk offer the possibility that the supplementation of the diet with fiber, butyrate, or probiotics containing butyrate-producing bacteria could reduce the risk of cardiovascular disease. Given the far-ranging implications of this, prospective and interventional clinical studies are certainly warranted to confirm the beneficial effects of butyrate.

Given the multifactorial nature of stroke, a risk stratification method was selected to establish the preliminary relationship between the gut microbiome and stroke risk in our study. We compared the microbial composition in the gut of participants at different risk levels of stroke. Our results also showed a gradual change in the gut microbiome profile from the LR to the MR to the HR group, indicating that this association was reliable. It is this approach that differentiates our study from previous studies of metabolic diseases such as diabetes mellitus, obesity, and even metabolic syndrome (MetS) (Ley et al., 2006; Sato et al., 2014; Lim et al., 2017; He et al., 2018a). MetS, which is characterized by three to five cardiovascular risk factors, including abdominal obesity, hypertension, hyperglycemia, and dyslipidemia, and which is associated with chronic low-grade inflammation leading to the development of cardiovascular disease (Esposito et al., 2004), is strikingly similar to the physiological state that confers a high risk of stroke. Indeed, a systematic review and meta-analysis demonstrated that MetS is closely linked with a high risk for stroke (Mottillo et al., 2010). MetS patients exhibit increased levels of *Lactobacillus* and decreased levels of *Bifidobacterium* in the gut (Lim et al., 2017), both of which generate lactate, which certain gut bacteria can then ferment into butyrate (Bourriaud et al., 2005; Bertacco et al., 2017). It is interesting to note that the levels of *Lactobacillus* and *Bifidobacterium* both more enriched in the HR group in our study. We thus hypothesize that the abundance of lactate-producing bacteria represents an attempt to compensate for the loss of butyrate-producing bacteria within the gut. It is possible that decreasing butyrate acts as a signal for lactate producers to proliferate and increase lactate production in order to shift the equilibrium toward the synthesis of butyrate. Complete elucidation of the mechanism relating lactate- and butyrate-producing gut bacteria, however, will obviously require further study.

Previous clinical studies of Chinese and German acute stroke patients described an increase in the abundance of Proteobacteria and *Megasphaera* and the reduction of the abundance of *Faecalibacterium* in the former (Yin et al., 2015), and an increase in Bifidobacteriaceae and Enterobacteriaceae and decrease in habitual bacteria such as *Roseburia* and *Faecalibacterium* in the latter (Swidsinski et al., 2012). By contrast, only *Lactobacillus* increased in a later study involving Japanese stroke patients (Yamashiro et al., 2017). Although our observation that the microbial composition of the participants judged to be at a high risk for stroke in this study was similar to that of acute stroke patients is intriguing, long-term prospective studies will be needed to confirm whether the prestroke condition of the gut microflora has any actual bearing on stroke outcome.

Medication use is very common in elderly adults, especially those with more than a 1-year course of hypertension, diabetes mellitus and dyslipidemias. Thus, it is important to make sure that analyses involving elderly adults are not confounded by drug use. In our study, we separately compared the gut microbial characteristics of different risk groups with or without medication use. The results revealed that the gut microbiota composition of individuals who were not taking medicine was similar to that of the total participants. The HR group was characterized by pathogenic bacteria, and the LR group had high levels of butyrate-producing bacteria. The gut microbiota composition of individuals who were taking medicine showed a similar pattern of gut microbial composition to those who were not taking medicine, suggesting that medication use might not alter the gut microbial characteristics of different risk groups. Previous study revealed that metformin intervention significantly increased *Escherichia* and decreased *Intestinibacter* abundance in the gut microbiota of DM patients (Forslund et al., 2015), but an *in-vitro* study got a negative result, and none of 40 bacteria strains was suppressed (Maier et al., 2018). Acarbose is recommended in clinical practice as an alternative to Metformin for first line treatment of DM patients. Acarbose treatment was demonstrated to deplete *Bacteroides* in the gut microbiota in both *in-vivo* and *in-vitro* experiment (Gu et al., 2017), but only found to increase the relative abundances of *Lactobacillus* and *Bifidobacterium* in the gut microbiota *in vitro* (Maier et al., 2018). In that *in-vitro* study, anti-commensal activity of human-targeted drug were also screened. Results found that several antihypertensive drugs could suppressed the growth of commensal bacteria (e.g., *Roseburia, Ruminococcus*) (Maier et al., 2018). Further *in vivo* studies to investigate the association between medicine, disease control and gut microbial modulation are still needed.

The risk stratification method used in our study was derived from the Stroke Risk Scorecard, which was also popularly recommended to stroke risk screening by National Stroke Association (http://www.stroke.org/stroke-resources/resource-library/stroke-risk-scorecard). Based on this, we tried to understand the relationship between gut microbiome alterations and the overall stroke risk, and the results suggested that gut microbiota might be a new risk factor for stroke. Although the findings of our research have important clinical implications, they should be interpreted with caution due to the small sample size and study limitations. In this cross-sectional study, it was not possible for us to determine the causal relationship between intestinal dysbiosis and stroke risk. Furthermore, metabolic factors are the main risk factors for stroke, and our criteria for risk stratification were based on the number of risk factors, which determines that the high risk of stroke always coexists with metabolic diseases. There are totally 141 participants in the present study, among which 94 participants coexist more than 1 risk factor. Taking diabetes as an example, there are only one individual with diabetes but free from all other risk factors. Thus, it is very difficult for the present study to disentangle the relative contribution of each risk factor to its relationship with the microbiome. It is interesting to investigate the relative contribution of the different risk factors to risk-associated differences in the microbiome, but a large-scale population-based study that recruited participants with separated risk factors is needed.

In summary, we found that a higher risk of stroke is associated with the enrichment of opportunistic pathogens, low abundance of butyrate-producing bacteria, and reduced concentrations of fecal butyrate. Based on cross-sectional data from our small sample, our study preliminarily explored the association between gut microbiota and long-term stroke risk, raising the possibility that the microbiome might be a novel risk factor of stroke. Additional multicenter studies with a larger sample are needed to validate our findings, as well as to verify if our findings are confined to a specific region or are general (He et al., 2018b).

## Data Availability

Data are available from the European Nucleotide Archive (https://www.ebi.ac.uk/ena/) at accession number PRJEB29939. All metadata analyzed in this study are included in Table S3.

## Ethics Statement

The study was conducted according to the principles of the Declaration of Helsinki and received approval from the Ethical Review Committee of Nanfang Hospital, Southern Medical University (approval letter number: NFEC-2016-148). Written informed consent was obtained from all study participants by neurologists prior to data and sample collection.

## Author Contributions

XZ and XG wrote the manuscript. CY and CT performed the stroke risk evaluations and collected the stool samples from all participants. YP, QW, and JZ designed and monitored the quality of the study. GX and XZ performed the data processing and statistical analysis. RX and CT detected the concentration of SCFAs in the fecal sample. JY, SP, YH, and HZ oversaw the manuscript revision process.

### Conflict of Interest Statement

The authors declare that the research was conducted in the absence of any commercial or financial relationships that could be construed as a potential conflict of interest.
