# Supplementary material for: Higher Risk of Stroke Is Correlated With Increased Opportunistic Pathogen Load and Reduced Levels of Butyrate-Producing Bacteria in the Gut
Source: Front Cell Infect Microbiol. 2019 Feb 4;9:4. doi: 10.3389/fcimb.2019.00004 (PMC6369648; doi:10.3389/fcimb.2019.00004)
Supplement: Supplementary file 1 [file Table_1.DOCX]

**Supplementary Method**

**Table S1. GC/MS Analytical method.**

| Parameter | Value |
| --- | --- |
| GC/MS System | Agilent 7890B GC/5977B MSD |
| Column | HP-FFAP 30 m × 0.25 mm, 0.25 μm (p/n 19091F-433) |
| Column flow | 1.0 mL/min |
| Liner | Agilent liner, split, single taper, wool, deactivated (p/n 5183-4711) |
| Injection mode | Split (30:1) |
| Injection temperature | 230 °C |
| Oven temperature | 90°C (1.0min), 15°C/min to 120 °C (0.5min),  15℃/min to 150℃ (0.5min),  15℃/min to 180℃ (1.0min). |
| Transfer line temperature | 230 °C |
| MS mode | SIM |
| SIM ions m/Z | 43,45,60,73,74,88 |
| Ion source | 5977 Inert Ion source Assy (p/n G3870-67700) |
| Ion source temperature | 230 °C |
| Quad. Temperature | 150 °C |
| GC/MS Data Acquisition | MassHunter GC/MS Acquisition Ver B.07.05.2479 |
| GC/MS Data Quantitative Analysis | MassHunter Workstation Software/ Quantitative Analysis Ver B.08.00 |

**Table S2. List of analytes, their retention times, and quantification ions.**

| No. | Compound | RT (min)* | m/z |
| --- | --- | --- | --- |
| 1 | Acetic acid | 4.419 | 43 (45, 60) |
| 2 | Propionic acid | 5.166 | 74 (73, 45) |
| 3 | Isobutytic acid | 5.405 | 43 (73) |
| 4 | Butyric acid | 5.954 | 60 (73) |
| 5 | Isovaleric acid | 6.340 | 60 (43, 45) |
| 6 | Valeric acid | 6.978 | 60 (73) |
| 7 | 2,2-dimethylbutyric acid | 7.161 | 88 (73, 43) |

* Retention times of analytes were based on standard series std2-1.

**Supplementary Figure**

Figure S1. Microbial analysis with a normalization of 6,000 sequences among the LR (n=41, green), MR (n=39, blue), and HR (n=31, red) groups. (A-D) Alpha diversity among the three groups. Average relative abundance of dominant microbes among the LR, MR, and HR groups at the phylum level (E) and family level (F), with each color representing a taxon. (G) Significantly discriminative taxa between the LR and HR groups were determined using linear discriminant analysis effect size (LEfSe) analysis. (H) Beta diversity comparison among the three groups. Principal coordinates analysis (PCoA) based on Bray–Curtis distance was used to illustrate the variations between the three groups. LR, low-risk group; MR, medium-risk group; HR, high-risk group; PD, phylogenetic diversity.

Figure S2. Microbial analysis of DADA2 among the LR (n=51, green), MR (n=52, blue), and HR (n=36, red) groups. (A-D) Alpha diversity among the three groups. Average relative abundance of dominant microbes among the LR, MR, and HR groups at the phylum level (E) and family level (F), with each color representing a taxon. (G) Significantly discriminative taxa among the LR, MR and HR groups were determined using linear discriminant analysis effect size (LEfSe) analysis. (H) Beta diversity comparison among the three groups. Principal coordinates analysis (PCoA) based on Bray–Curtis distance was used to illustrate the variations between the three groups. LR, low-risk group; MR, medium-risk group; HR, high-risk group; PD, phylogenetic diversity.

Figure S3. The relative abundance of dominated taxa in the gut of each sample among the LR (n=51, green), MR (n=54, blue), and HR (n=36, red) groups at the phylum level. Different colors represent different taxa. The height of each color on the Y-axis represents the abundance of different bacteria in each sample on the X-axis. LR, low-risk group; MR, medium-risk group; HR, high-risk group. P, participant.

Figure S4. The relative abundance of dominated taxa in the gut of each sample among the LR (n=51, green), MR (n=54, blue), and HR (n=36, red) groups at the family level. Different colors represent different taxa. The height of each color on the Y-axis represents the abundance of different bacteria in each sample on the X-axis. LR, low-risk group; MR, medium-risk group; HR, high-risk group. P, participant.

Figure S5. Differences in the composition of gut microbial communities among the LR (n=44, green), MR (n=12, blue), and HR (n=7, red) groups without any medicines used. (A-D) Alpha diversity among the three groups. Average relative abundance of dominant microbes among the LR, MR, and HR group at the phylum level (E) and family level (F), with each color representing a taxon. (G) Significantly discriminative taxa among the LR, MR, and HR group were determined using linear discriminant analysis effect size (LEfSe) analysis. LR, low-risk group; MR, medium-risk group; HR, high-risk group. PD, phylogenetic diversity.

Figure S6. Differences in the composition of gut microbial communities among the LR (n=7, green), MR (n=42, blue), and HR (n=29, red) groups with at least one medicine used. (A-D) Alpha diversity among the three groups. Average relative abundance of dominant microbes among the LR, MR, and HR group at the phylum level (E) and family level (F), with each color representing a taxon. (G) Significantly discriminative taxa among the LR, MR, and HR group were determined using linear discriminant analysis effect size (LEfSe) analysis. LR, low-risk group; MR, medium-risk group; HR, high-risk group. PD, phylogenetic diversity.
