# Supplementary figures and images for: Higher Risk of Stroke Is Correlated With Increased Opportunistic Pathogen Load and Reduced Levels of Butyrate-Producing Bacteria in the Gut
Source: Front Cell Infect Microbiol. 2019 Feb 4;9:4. doi: 10.3389/fcimb.2019.00004 (PMC6369648; doi:10.3389/fcimb.2019.00004)

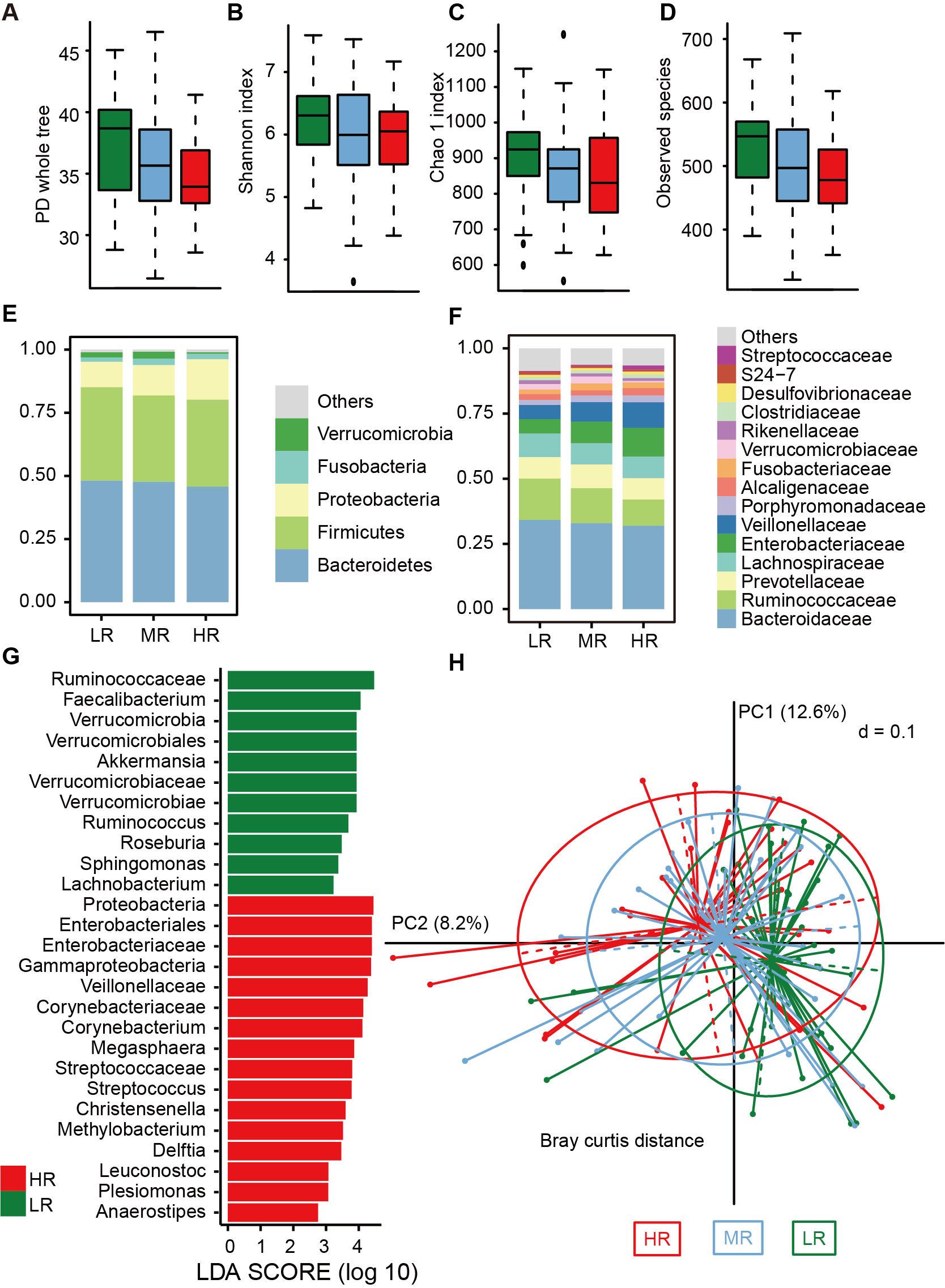

Supplement: Supplementary file 3 [file Image_1.TIF]

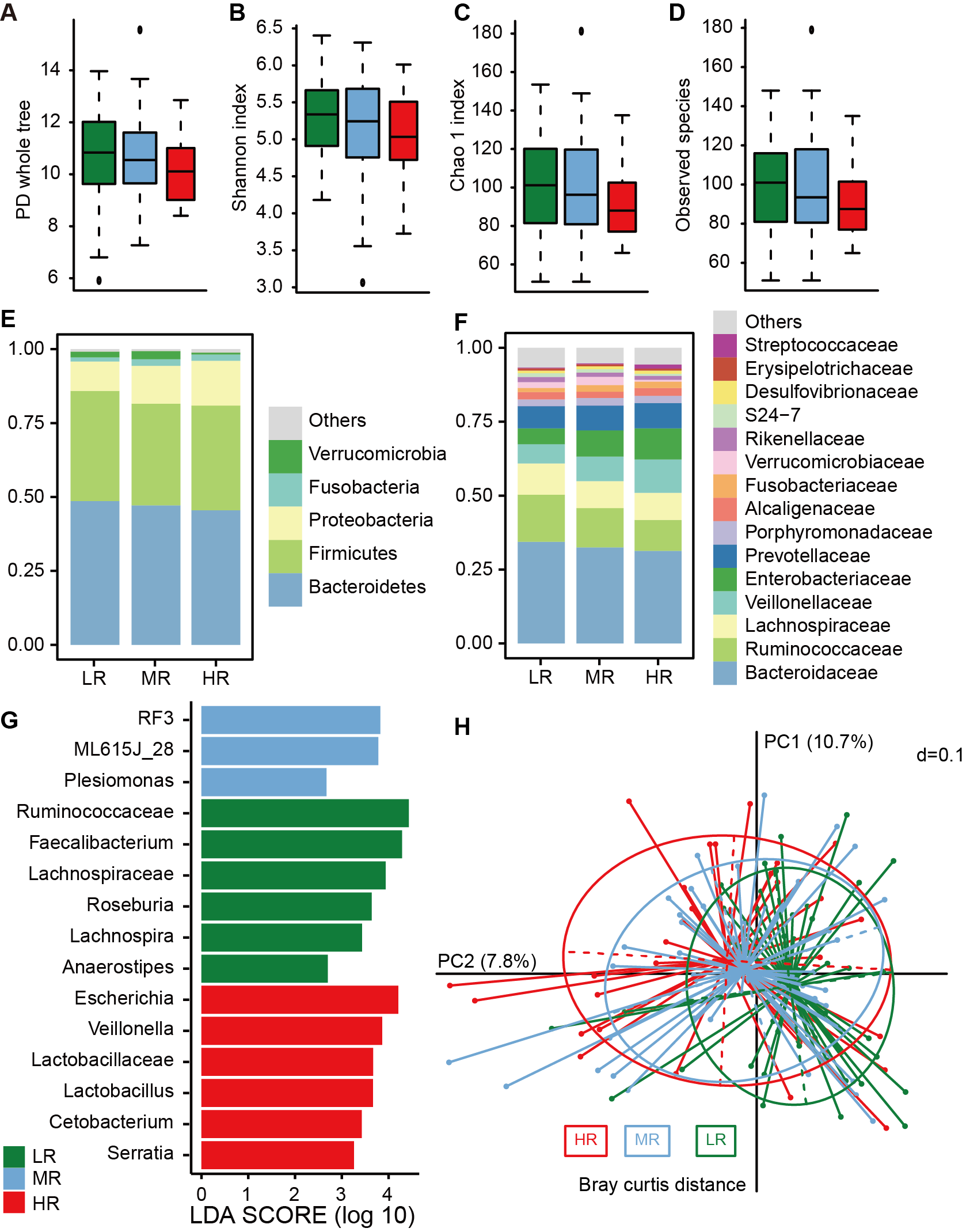

Supplement: Supplementary file 4 [file Image_2.TIF]

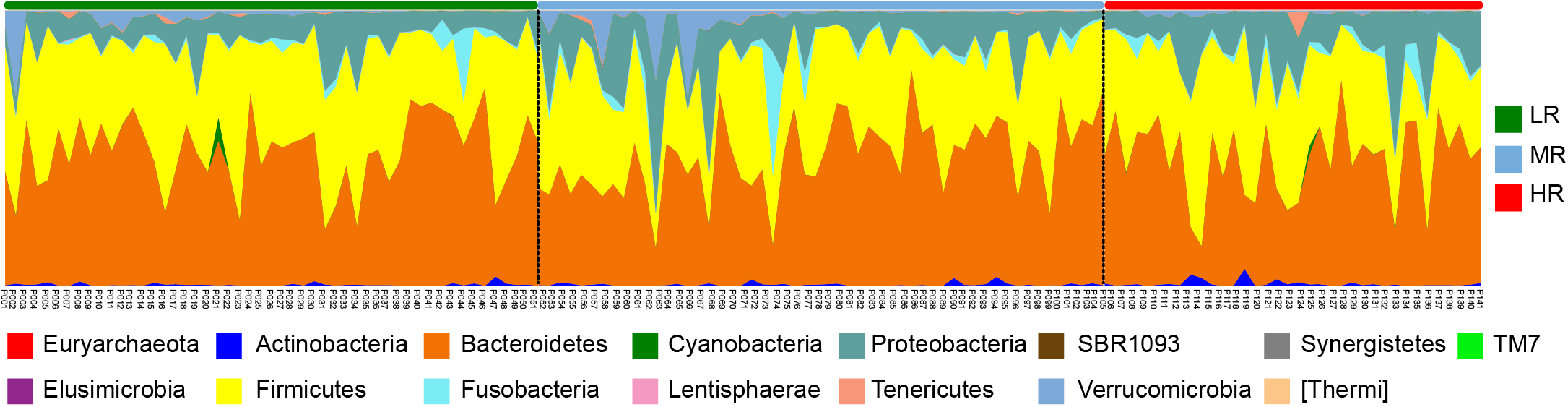

Supplement: Supplementary file 5 [file Image_3.TIF]

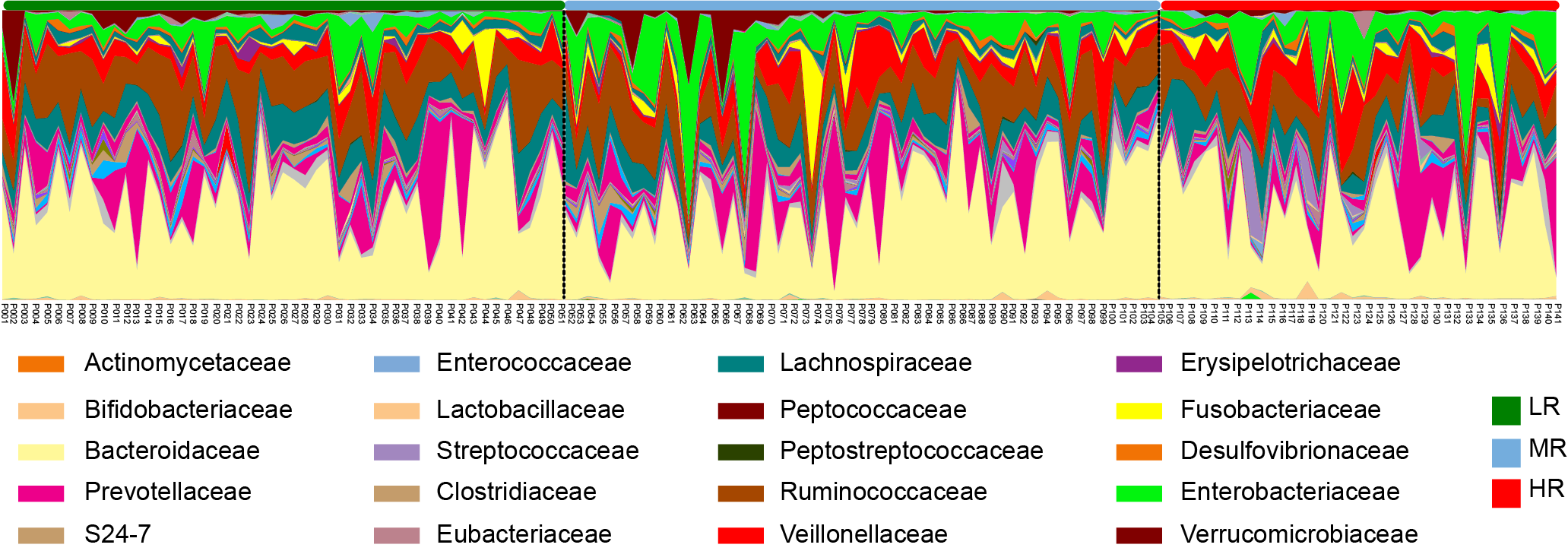

Supplement: Supplementary file 6 [file Image_4.TIF]

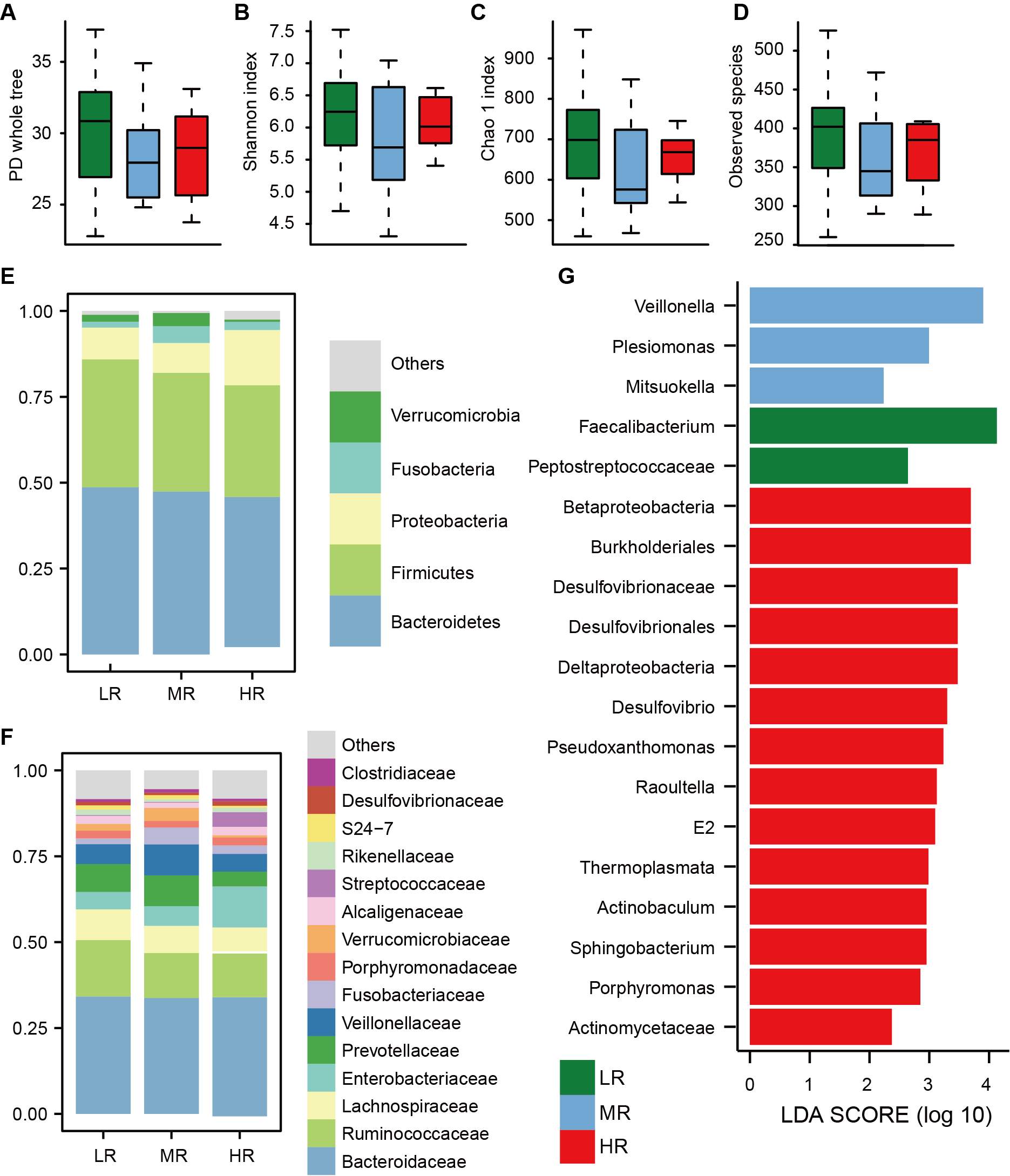

Supplement: Supplementary file 7 [file Image_5.TIF]

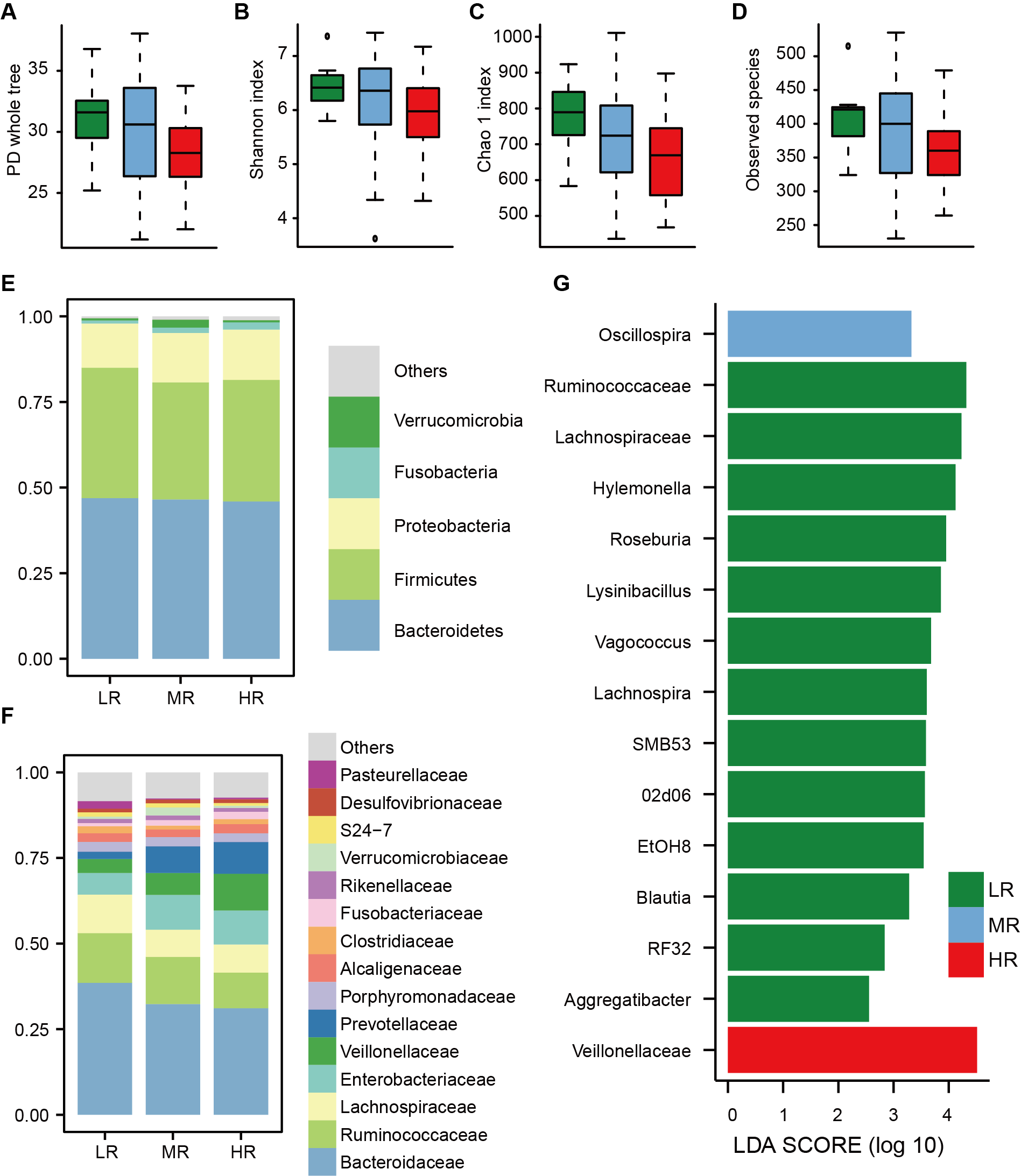

Supplement: Supplementary file 8 [file Image_6.TIF]
